# RETRACTED ARTICLE: Predictive models for the occurrence and lymph node metastasis of papillary thyroid carcinoma with regional risk heterogeneity

**DOI:** 10.1038/s41746-026-02649-8

**Published:** 2026-04-18

**Authors:** Zhigang Zhang, Hongyu Liu, Zheng Zhao, Guoyu Tan, Yang Zhao, Xun Liu

**Affiliations:** 1 Departmen of Emergency Medicine, Shengjing Hospital of China Medical University, Shenyang, Liaoning China; 2 Lun Lun Research Institute of AI FOR SCIENCE, Beijing Diji Technology Co., Ltd., Beijing, China; 3 Department of General Surgery, Shengjing Hospital of China Medical University, Shenyang, Liaoning China

**Keywords:** Biomarkers, Cancer, Computational biology and bioinformatics, Oncology

The authors have retracted this article.

Following publication, the authors identified major methodological errors, including issues related to the sequence of multi-stage feature selection and multi-centre batch correction. After correcting these errors, they found that the appropriate evaluation metrics and downstream biological interpretations differed substantially from those reported in the published article. In addition, the model's performance and its ability to generalise across datasets were considerably lower than originally presented. As a result, the conclusions of the article are no longer supported by the corrected analyses and are therefore not reliable.

Zhigang Zhang, Hongyu Liu, Guoyu Tan, Yang Zhao and Xun Liu agree with this retraction. Zheng Zhao did not respond to any correspondence from the Publisher regarding this retraction.

## Supplementary information

