# Supplementary material for: RETRACTED ARTICLE: Predictive models for the occurrence and lymph node metastasis of papillary thyroid carcinoma with regional risk heterogeneity
Source: NPJ Digit Med. 2026 Apr 18;9:652. doi: 10.1038/s41746-026-02649-8 (PMC13507310; doi:10.1038/s41746-026-02649-8)
Supplement: Supplementary file 1 [file 41746_2026_2649_MOESM1_ESM.pdf]

Published in partnership with Seoul National University Bundang Hospital

https://doi.org/10.1038/s41746-026-02649-8

Predictive models for the occurrence and lymph node metastasis of papillary thyroid carcinoma with regional risk heterogeneity

Check for updates

Zhigang Zhang[1,4], Hongyu Liu[1,4], Zheng Zhao[1,4], Guoyu Tan[2], Yang Zhao[3] ✉ & Xun Liu[3] ✉

Papillary thyroid carcinoma (PTC) is the most common thyroid malignancy, and cervical lymph node metastasis significantly impacts patient prognosis. This study aimed to develop interpretable artificial intelligence models based on transcriptomics to predict PTC occurrence and cervical lymph node metastasis, while exploring the heterogeneity of risk factors across different regions. We obtained 419 samples from the GEO database, originating from Asia, Europe, and America, comprising 158 normal samples, 203 PTC samples, and 58 metastatic samples. After comparing multiple machine learning algorithms, deep neural networks (DNN) demonstrated superior performance and were used to construct the PTC diagnostic and metastasis predictive models. The optimized PTC diagnostic model achieved an AUC of 0.987 with an accuracy of 0.945, while the PTC metastasis predictive model reached an AUC of 0.998 with an accuracy of 0.987. Model interpretation using SHapley Additive exPlanations (SHAP) and Kolmogorov-Arnold Networks (KAN) methods identified SYT1, REN, CNTN5, and ADAM12 as critical features for PTC diagnosis, whereas COL9A1, CYP4F3, and GAD1 were key predictors for PTC metastasis. Stratification analysis revealed regional differences in risk factors for PTC occurrence, while factors promoting PTC metastasis exhibited commonalities across different regions. Pathway enrichment analysis indicated that regulation of hormone levels and cell population proliferation were common pathways involved in both PTC occurrence and metastasis. Finally, we developed online predictive platforms based on the Streamlit framework to facilitate transparent model exploration and risk estimation. These tools are publicly accessible research-use prototypes intended for model demonstration and interpretability visualization. Because they require standardized gene-expression inputs and have not undergone prospective clinical validation, they should not be used as standalone tools for clinical diagnosis, risk stratification, or treatment decision-making. Overall, our findings identify candidate transcriptomic markers associated with PTC occurrence and lymph node metastasis and provide a basis for future translational evaluation.

Thyroid cancer has emerged as one of the most prevalent endocrine malignancies globally, with papillary thyroid carcinoma (PTC) accounting for approximately 85–90% of all cases[1]. Over the past three decades, the incidence of PTC has increased by 3–5% annually, a trend largely attributed to advancements in ultrasound and imaging technologies that enhance the detection of small thyroid nodules (≤1 cm)[2]. However, this diagnostic progress has also led to a clinical dilemma: while early identification improves outcomes, it raises concerns about overdiagnosis and over-treatment of indolent lesions. Distinguishing benign from malignant nodules remains a critical challenge, as unnecessary interventions carry risks

[1]Departmen of Emergency Medicine, Shengjing Hospital of China Medical University, Shenyang, Liaoning, China. [2]Lun Lun Research Institute of AI FOR SCIENCE, Beijing Diji Technology Co., Ltd., Beijing, China. [3]Department of General Surgery, Shengjing Hospital of China Medical University, Shenyang, Liaoning, China. [4]These authors contributed equally: Zhigang Zhang, Hongyu Liu, Zheng Zhao. ✉e-mail: treezy@163.com; liuxun@sj-hospital.org

of complications, including recurrent laryngeal nerve injury and hypoparathyroidism[3]. Traditional diagnostic methods, including fine-needle aspiration biopsy (FNAB), frequently produce inconclusive results, with approximately 20–30% of cases falling into the indeterminate Bethesda III or IV categories[4]. Meanwhile, current molecular diagnostic markers, such as BRAF V600E, RAS, and TERT promoter mutations, offer limited insights into tumor behavior. Their sensitivity and specificity for predicting aggressive phenotypes, particularly lymph node metastasis (LNM), are still not ideal[5]. Therefore, accurate prediction of PTC occurrence and lymph node metastasis is crucial for developing individualized treatment strategies and improving patient outcomes.

Despite the generally favorable prognosis of PTC, with 10-year survival rates exceeding 90%, the management of cervical LNM remains controversial[6]. Prophylactic central lymph node dissection, once widely adopted, is increasingly scrutinized due to its association with surgical morbidity without clear survival benefits. Molecular markers such as TERT promoter mutations and BRAF/RAS co-mutations have been linked to higher risks of LNM and disease recurrence, yet their predictive power is inconsistent across populations[7]. Current guidelines rely heavily on clinicopathological features, such as tumor size and extrathyroidal extension, which lack granularity for personalized surgical planning[8]. Consequently, developing novel biomarkers and predictive models to improve the accuracy of PTC diagnosis and metastasis prediction has become a research focus.

Recent years, machine learning (ML) has shown promise in refining molecular diagnostics and prognostication for PTC. Studies leveraging transcriptomic, proteomic, and radiomic data have demonstrated the potential of ML models to predict LNM and recurrence risk[9]. However, existing approaches face critical limitations: (1) models trained on single-omics data often fail to capture the multidimensional nature of tumor biology; (2) "black-box" algorithms, such as deep neural networks, lack interpretability, hindering clinical adoption; and (3) validation cohorts frequently lack diversity in ethnicity and geographic representation, compromising generalizability[10].

The interpretability of artificial intelligence models is crucial for medical applications[11]. Interpretable AI models not only provide accurate predictions but also explain the decision logic behind the predictions, helping physicians understand the reasoning process and enhancing trust in the prediction results[12]. The emergence of novel interpretable AI methods such as SHAP (SHapley Additive exPlanations) and KAN (Kolmogorov-Arnold Networks) in recent years has provided new approaches for constructing transparent and reliable medical prediction models[13–15].

This study aims to develop interpretable artificial intelligence models for PTC diagnosis and lymph node metastasis prediction based on transcriptomic data from multiple regions worldwide, and to explore the heterogeneity of risk factors across different regions. Through this research, we expect to not only provide accurate tools for PTC diagnosis and metastasis prediction but also reveal the molecular mechanisms underlying PTC occurrence and metastasis, and explore region-specific risk factors, thereby providing scientific evidence for individualized precision medicine.

Results

Model development and feature screening

In this study, a diagnostic model was first constructed using a DNN algorithm with both the full-feature set and a reduced 20-feature set. The 20-feature model achieved comparable—and even superior—diagnostic performance relative to the full-feature model, with an AUC of 0.987 (95% CI: 0.969–0.998), outperforming the full-feature model whose AUC was 0.921 (95% CI: 0.858-0.971). Other evaluation metrics of the 20-feature DNN model also demonstrated excellent diagnostic ability, including an accuracy of 0.945, a precision of 0.982, and an F1 score of 0.949 (Fig. 1A and Supplementary Figure S1A). Based on the optimized 20-feature set, five machine-learning algorithms—DNN, GB, Bagging, AdaBoost, and LR—were subsequently employed for comparative analysis. As shown in Fig. 1B, DNN exhibited the best diagnostic performance, achieving an AUC of 0.987, an accuracy of 0.945, a precision of 0.982, an F1 score of 0.949, and a recall of 0.918 (Fig. 1B and supplementary Table 1).

To construct the PTC metastasis predictive model, both full-feature and 20-feature models were first developed using the DNN algorithm. The 20-feature DNN model demonstrated excellent predictive performance, yielding an AUC of 0.998 (95% CI: 0.992–1.000), an accuracy of 0.987, a precision of 1.000, a recall of 0.984, and an F1 score of 0.992 (Fig. 1C and Supplementary Figure S1B), outperforming the full-feature model and indicating that the reduced feature set provided robust predictive capability. Based on the optimized 20-feature set, five machine learning algorithms—including DNN, Bagging, AdaBoost, KNN, and Decision Tree—were subsequently applied for model comparison. As shown in Fig. 1D and Supplementary Table 2, DNN exhibited the best performance among the five algorithms, achieving an AUC of 0.998, an accuracy of 0.987, a precision of 1.000, a recall of 0.984, and an F1 score of 0.992.

External Validation of Model Generalizability

To evaluate the generalizability of our models, we performed external validation using the TCGA-THCA cohort. The diagnostic model was validated in a dataset containing 505 tumor samples and 59 normal controls. The PTC diagnostic model demonstrated excellent generalization performance, achieving an ROC AUC of 0.995 (95% CI: 0.990–0.999, Fig. 1E). For metastasis prediction, only eight PTC cases in TCGA-THCA were annotated with lymph-node metastasis, resulting in an extremely imbalanced dataset. To address this issue, we generated balanced datasets by randomly undersampling the 505 tumor samples to match the eight metastatic samples. Using three independently generated balanced datasets, the metastasis prediction model achieved the AUC values of 0.838 (95%CI: 0.609–1.000, Fig. 1F), 0.805 (95%CI: 0.531–1.000, Fig. 1G), and 0.807 (95% CI: 0.536–1.000, Fig. 1H), respectively. The model performance metrics were shown in Supplementary Figure S1C-S1D. These results provide preliminary support for the external applicability of the metastasis prediction model; however, the evidence remains limited because only eight metastatic TCGA-THCA cases were available and evaluation required repeated random undersampling.

Interpretable analysis with SHAP

Next, we used SHAP to interpret the optimal PTC diagnostic model and PTC metastasis predictive model. Figure 2A, B presented the contributions of features in PTC diagnosis. The feature importance rank revealed that SYT1, REN, CNTN5, ADAM12, HAPLN1, GABRA6, SCG3, GRIA3 and LIPC were the most influential factors for PTC diagnosis. Figure 2C, D revealed the important features for the prediction of PTC metastasis. COL9A1, CYP4F3, GAD1, PCOLCE2, ART4, KIF23, NLGN1, KCNC1, FOXD1 were identified to be the most important predictors for PTC metastasis.

Moreover, SHAP local interpretation illustrated how various features affected the predictive outcome for individual instance. SHAP force plot in Fig. 3A, B showed the diagnostic result of the very low risk of PTC (0.23) and the high risk of PTC (0.91). The positive contribution of SYT1, GRIA3, ADAM12 and REN pushed the diagnostic outcome to PTC (Fig. 3B). Figure 3C, D revealed the predictive outcome of PTC with no metastasis (0.02) and PTC with metastasis (0.98). The positive contribution of PCOLCE2, SLC16A7, ART4, CYP4F3 promoted the predictive result of PTC metastasis (Fig. 3D). The SHAP waterfall diagrams illustrated the decision-making process and feature contributions for individual samples (Fig. 3E, F). Figure 3E presents an example from the diagnostic model. The initial predicted value, f(x), was 0.559, with positive contributions from HAPLN1 (+ 0.18), REN (+ 0.09), PID1 (+ 0.06), and RELN (+ 0.05), and a negative contribution from GABRA6 (-0.04), resulting in a final predicted value of 0.985. The final prediction was PTC. Figure 3F presents an example from the metastasis prediction model. The initial predicted value, f(x), was 0.811, and after negative contributions from CYP4F3 (-0.45), CRISP2 (-0.44), and KCNC1 (-0.29), together with a positive contribution from HPGD (+ 0.58), the final predicted value was 0.002. The final prediction was non-metastatic PTC, indicating a very low risk of lymph node metastasis.

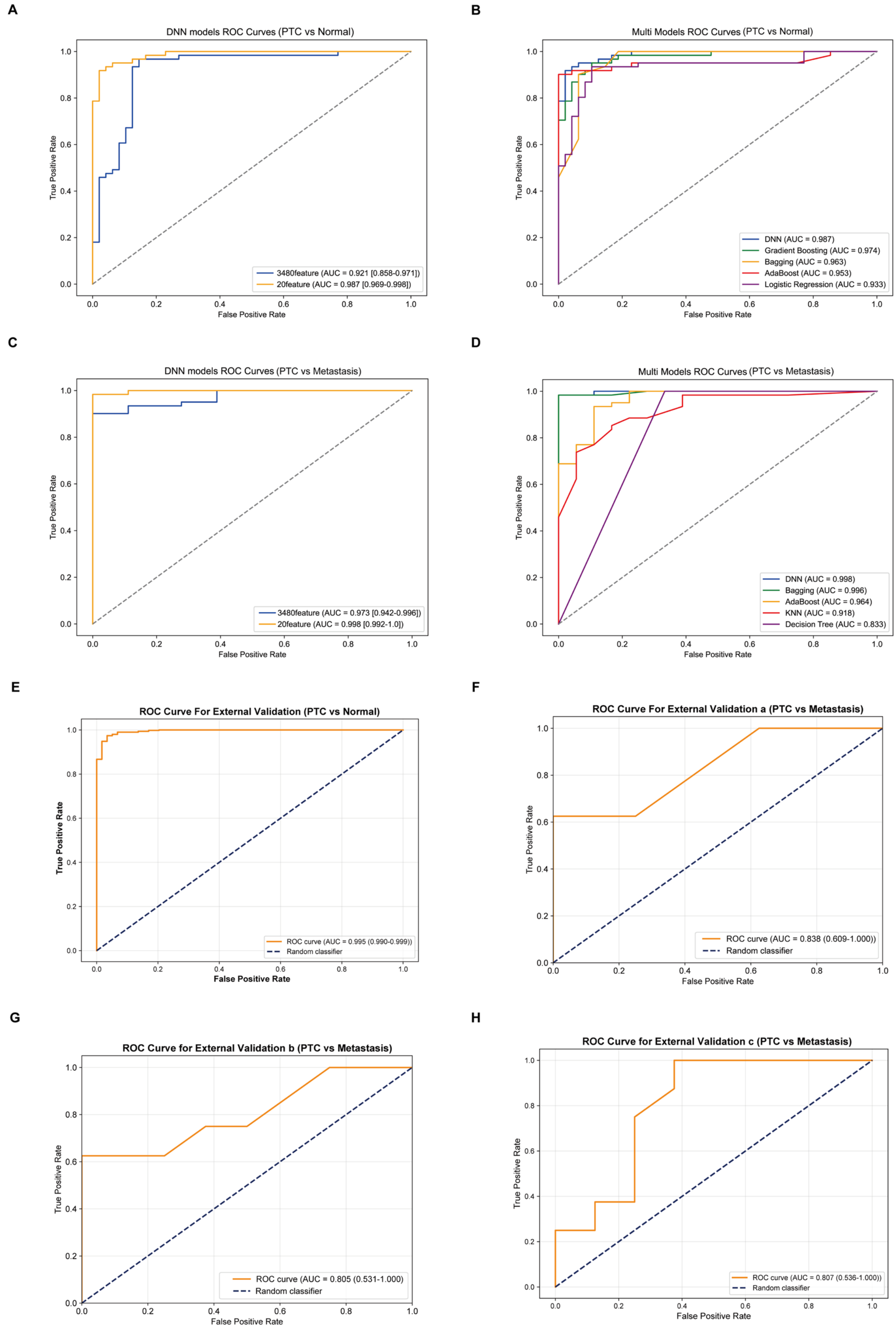

Fig. 1 | Model performance. A ROC curves of DNN model with different features for PTC diagnosis, **B** ROC curves of multiple algorithms for PTC diagnosis **C** ROC curves of DNN model with different features for PTC metastasis. **D** ROC curves of multiple algorithms for PTC metastasis prediction **E** External validation of PTC diagnostic model. **F** External validation a of PTC metastasis prediction. **G** External validation b of PTC metastasis prediction. **H** External validation c of PTC metastasis prediction.

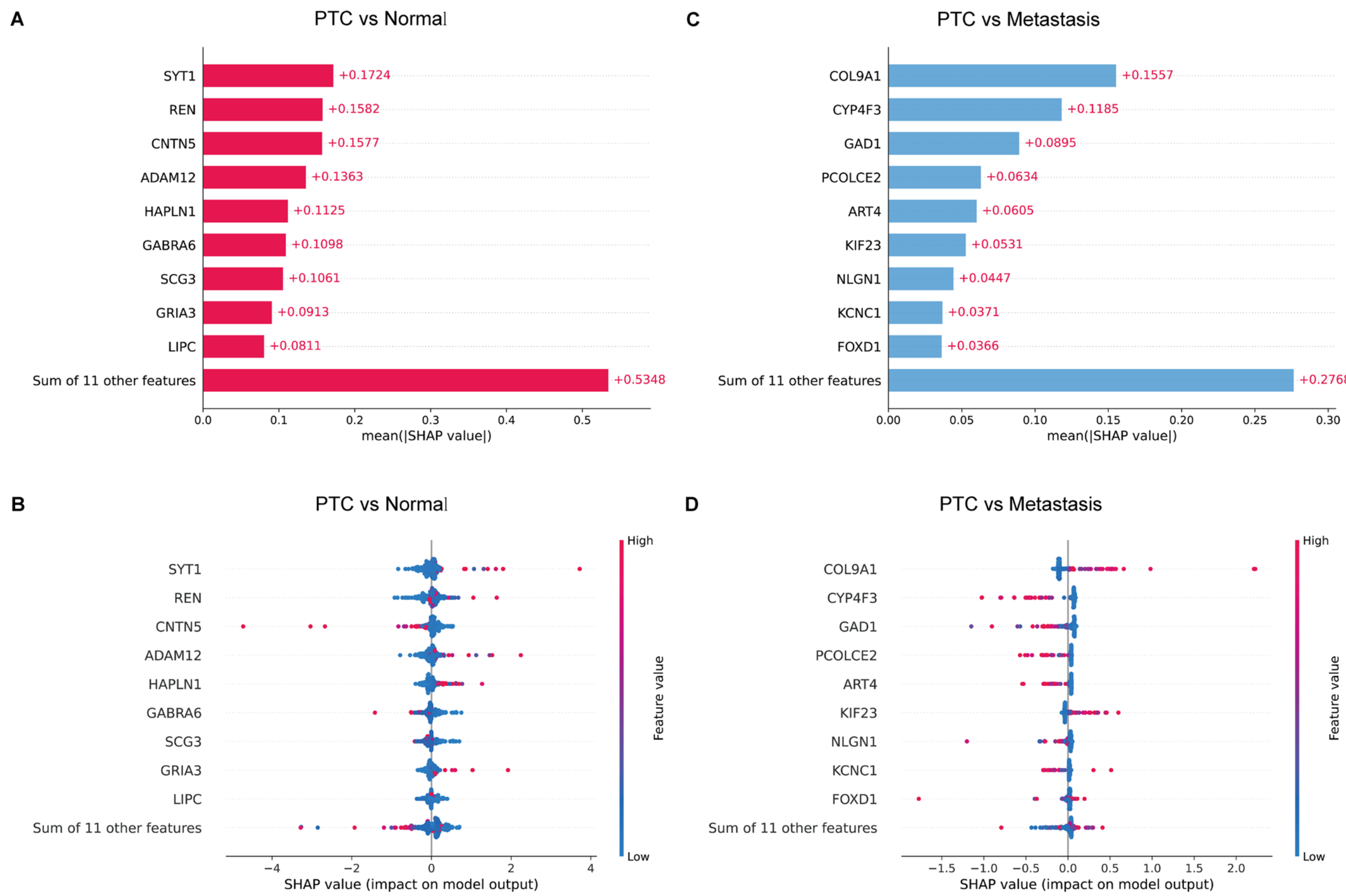

Fig. 2 | Global explanation of the model by SHAP. A, **B** Bar plot and beeswarm diagram showed the important features for PTC diagnosis. **C**, **D** Bar plot and beeswarm diagram showed the important features for PTC metastasis prediction.

Stratification analysis across distinct continents

To further investigate the heterogeneity of the pathogenic mechanisms of PTC occurrence and metastasis across different continents, we evaluated feature importance analysis of PTC diagnostic model and PTC metastasis predictive model in three continent-America, Asia and Europe, respectively. It was found that SYT1 was the most influential feature across three continents for PTC diagnosis (Fig. 4A–C). SYT1, CNTN5, ADAM12, and REN were identified the most important factors in PTC diagnostic models of Asia and Europe. Apart from SYT1, REN, and CNTN5, GABRA6 was the most important feature in America. These findings suggest that there are certain regional differences in the pathogenic factors of PTC occurrence. Interestingly, COL9A1, CYP4F3, and GAD1 were the most influential across three continents for PTC metastasis prediction, indicating that the pathogenic factors promoting PTC metastasis have commonalities between regions (Fig. 4D–E).

Interpretable analysis with KAN

Furthermore, the final model was explained by KAN. The PTC diagnostic model optimized by KAN showed an AUC of 0.94 (Fig. 5A). Network analysis provided a framework illustrating the relationships between key feature input and output nodes related to PTC diagnosis (Fig. 5B). KAN also presented the complex nonlinear relationships through mathematical expressions as shown in Supplementary Figure 2A. Consistent with the SHAP analysis, feature importance analysis indicated that SYT1, CNTN5 were of the significant importance for PTC diagnosis (Fig. 5C). The AUC of the PTC metastasis predictive model optimized by KAN was 0.90, demonstrating an excellent predictive performance (Fig. 5D). Network analysis showed an architecture between crucial features input and output nodes related to PTC metastasis (Fig. 5E). The mathematical expression for PTC metastasis prediction was shown in Supplementary Figure 2B. Feature importance analysis revealed CYP4F3 was the most important feature in PTC metastasis.

Online predictive platform

To improve the accessibility of the final models, web-based calculators for the PTC diagnostic model and the PTC metastasis predictive model were developed (Fig. 6A, B). The web links were as follows: https://ptc-normal.streamlit.app/ (PTC diagnosis) and https://ptc-metastasize.streamlit.app/ (PTC metastasis prediction). These online platforms provide accessible implementations of the final models and their interpretability outputs. They are intended as research-use prototypes for model demonstration and supplementary risk estimation, rather than as tools to replace established clinical diagnostic workflows. Because they require preprocessed standardized gene-expression inputs and have not undergone prospective real-world validation, they should not be used as standalone systems for clinical diagnosis, risk stratification, or treatment decision-making.

Pathway enrichment analysis

To further clarify the potential mechanism of PTC development and metastasis. Pathway enrichment analysis was conducted to explore the biological relevance of the features driving the predictive models. For the PTC diagnostic model, the full set of selected features was primarily enriched in pathways related to cell growth, cell–substrate junction organization, DNA-binding transcription factor binding, and protein serine/threonine/tyrosine kinase activity, as well as key oncogenic signaling pathways including PI3K/Akt and MAPK signaling. Notably, these enriched pathways are closely aligned with those involving classical PTC diagnostic biomarkers such as BRAF V600E, RAS, and TERT promoter mutations, supporting the biological plausibility of the diagnostic model (Supplementary Figure S3A). For the PTC metastasis prediction model, the full-feature set showed significant enrichment in pathways associated with cell–cell signaling by Wnt, cell–cell junction organization, ubiquitin-like protein ligase binding, and major tumor progression–related pathways

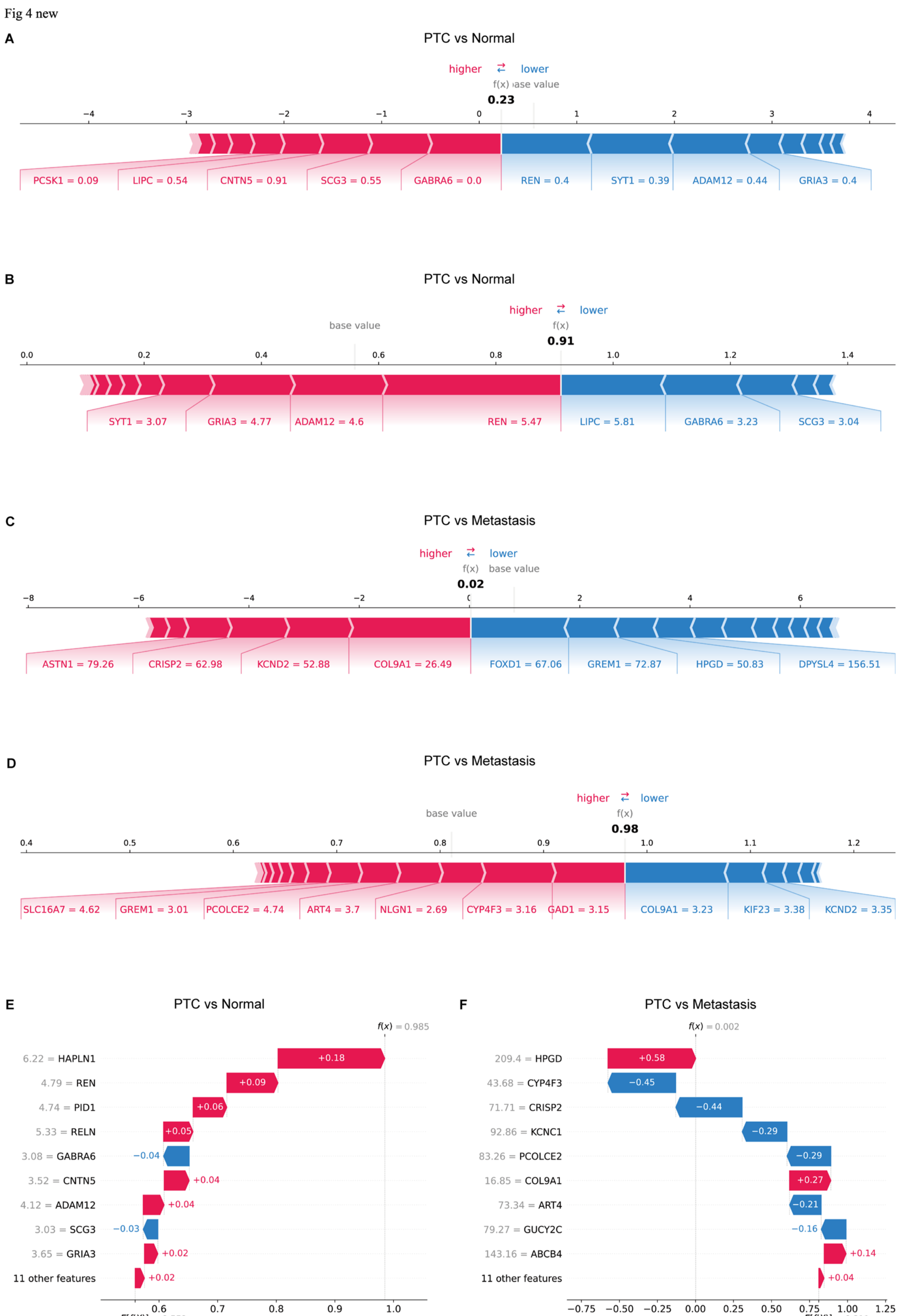

Fig. 3 | Local explanation of the model by SHAP. A, B Force plot showing the diagnostic outcome of normal or PTC. **C, D** Force plot showing the predictive outcome of PTC or metastasis. **E, F** Waterfall diagrams showing the contributions and the decision-making process of various features on model prediction.

including PI3K/Akt, MAPK, and Rap1 signaling. These enriched pathways correspond to biological processes known to influence tumor invasion, adhesion dynamics, and lymphatic dissemination, indicating that the metastasis model captures key mechanisms underlying metastatic potential (Supplementary Figure S3B).

Discussions

This study developed interpretable artificial intelligence models for PTC diagnosis and lymph node metastasis prediction based on transcriptomic data from multiple geographic regions and explored regional heterogeneity in associated risk factors. Our findings suggest that transcriptomics-based

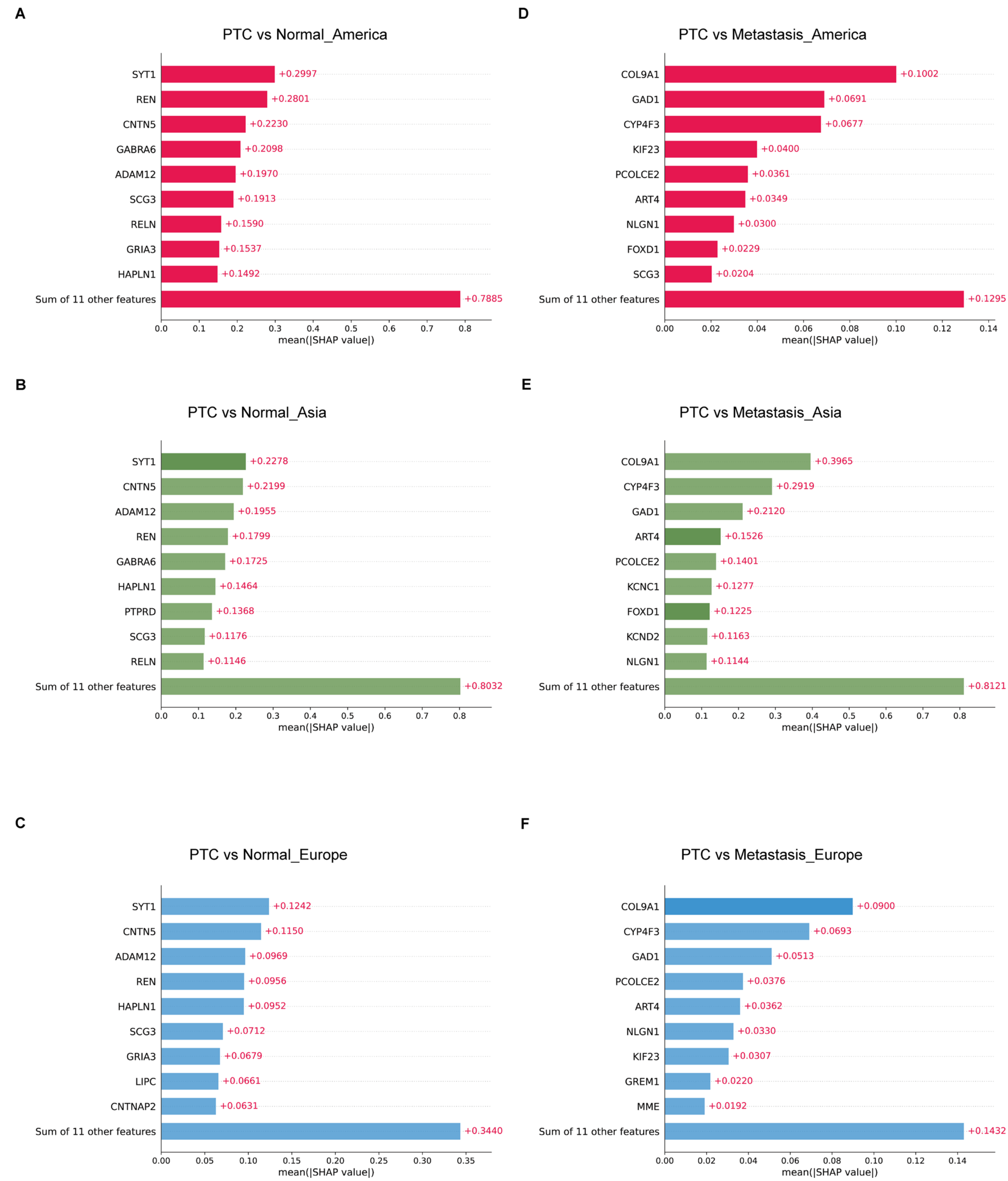

Fig. 4 | Stratification analysis across three continents. A–C PTC diagnostic model in America, Asia and Europe. **D–F** PTC metastasis predictive model in America, Asia and Europe.

interpretable models can achieve strong predictive performance in this dataset and highlight candidate molecular features associated with PTC occurrence and metastasis, thereby supporting future translational and mechanistic studies.

Both our PTC diagnostic model and metastasis predictive model demonstrated excellent performance, with the DNN algorithm outperforming other machine learning methods. After feature selection, the 20-feature DNN diagnostic model achieved an AUC of 0.987 and an accuracy of 0.945, while the metastasis predictive model reached an AUC of 0.998 and an accuracy of 0.987. These results compare favorably with previously reported prediction models based on clinical factors or single biomarkers, although direct cross-study comparison should be interpreted cautiously because of differences in cohorts, endpoints, and validation strategies[16–18], indicating that multi-gene feature combinations can more comprehensively capture the molecular characteristics of the disease and improve prediction accuracy.

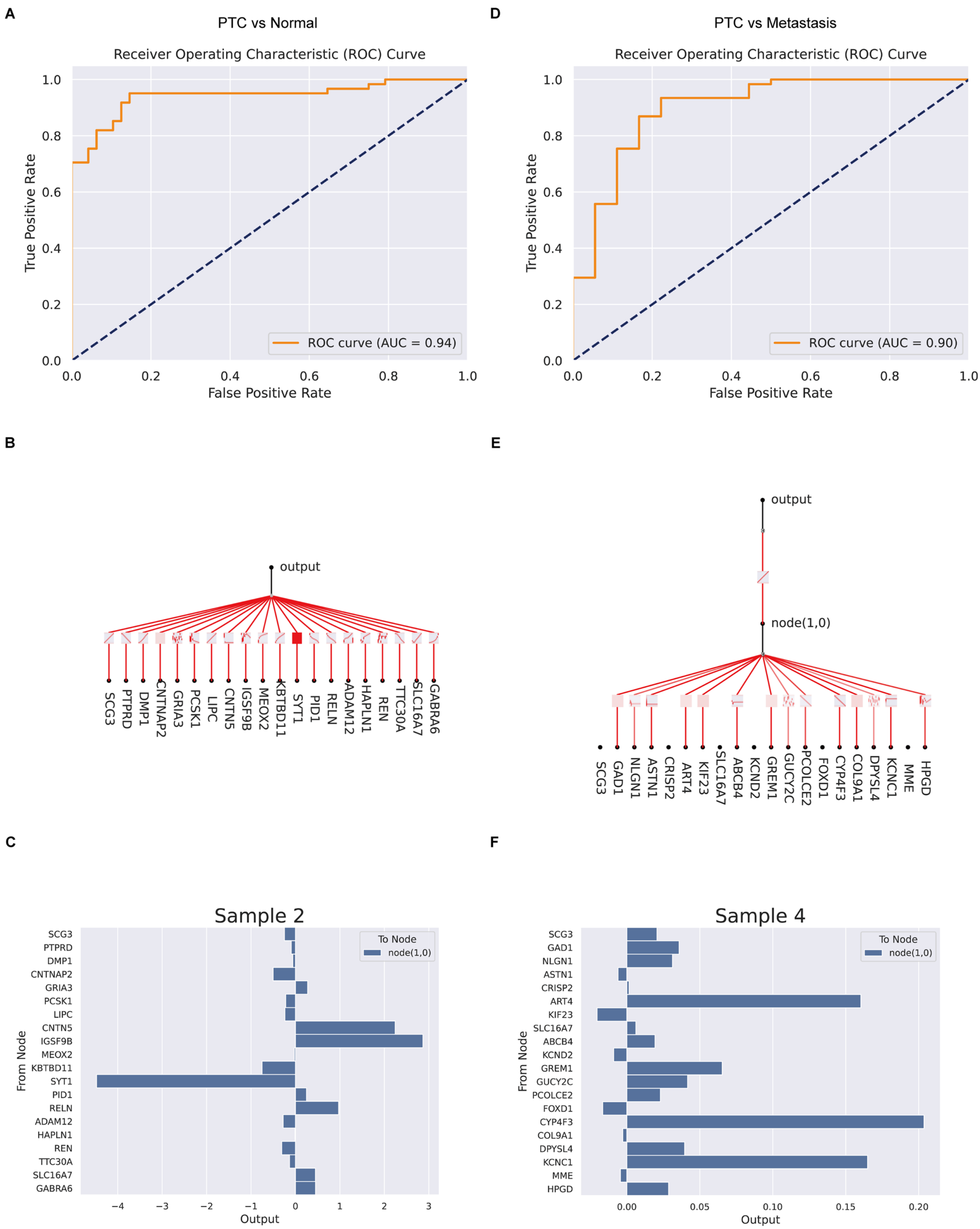

Fig. 5 | Kolmogorov-Arnold Networks (KAN). **A** ROC curve of PTC diagnosis model, **B** Network analysis of PTC diagnosis model, **C** Feature importance rank of PTC diagnosis model. **D**-**F** ROC curve, network analysis, feature importance rank of PTC metastasis predictive model.

Through SHAP and KAN analyses, we identified key genes for PTC diagnosis and metastasis prediction. SYT1, REN, CNTN5, and ADAM12 were determined to be important features for PTC diagnosis, which partially aligns with findings from previous studies. SYT1 (synaptotagmin 1) is involved in regulating neurotransmitter release and cellular secretion, and previous research has shown its aberrant expression in various cancers[19–22]. ADAM12 is a membrane-bound metalloprotease that has been demonstrated to play roles in promoting tumor cell proliferation, migration, and invasion[23–27]. For PTC metastasis prediction, COL9A1, CYP4F3, and GAD1 were identified as the most influential features. COL9A1 encodes the α1

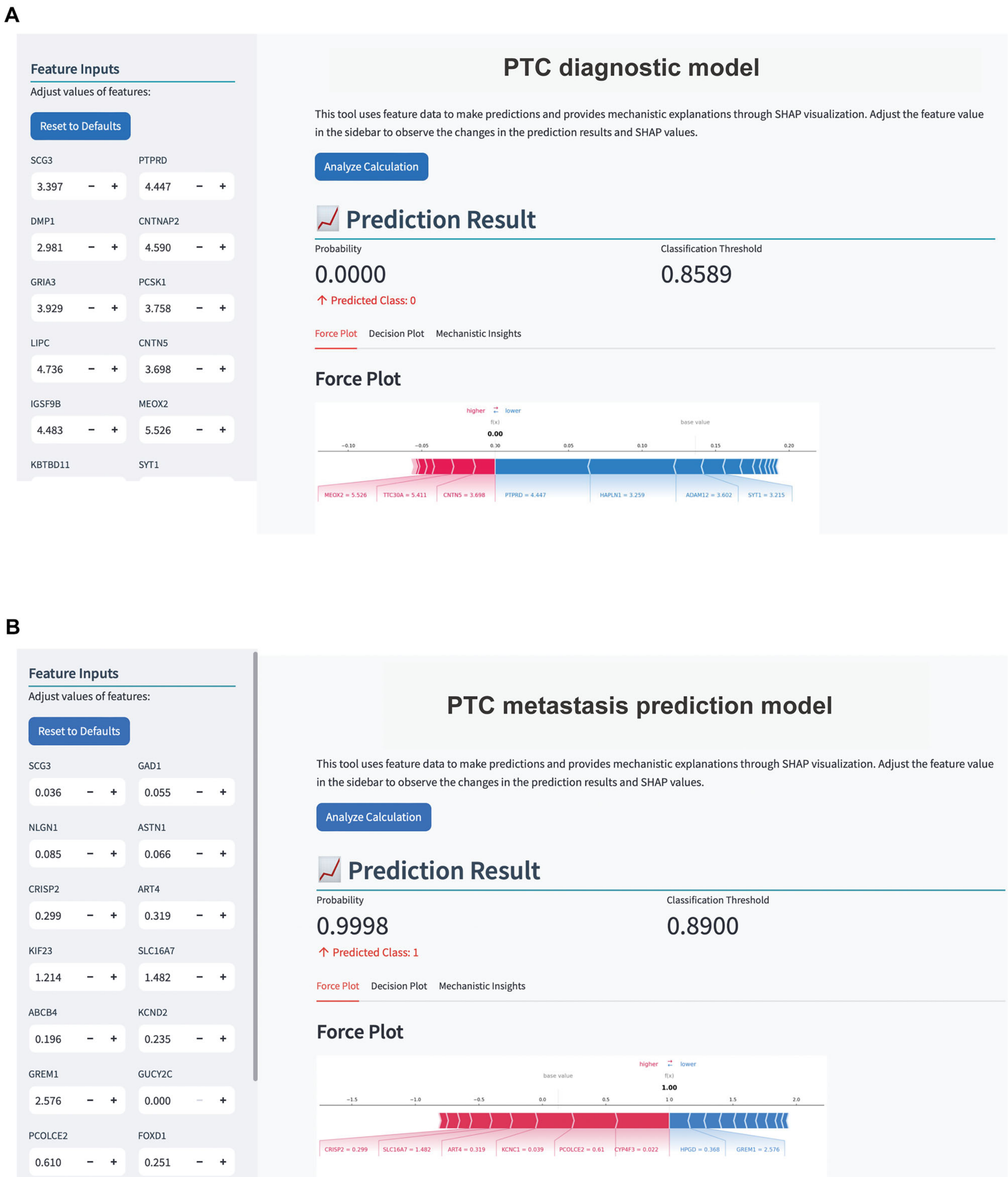

Fig. 6 | The representative image of online prediction platform. **A** PTC diagnosis model **B** PTC metastasis predictive model.

chain of type IX collagen, which participates in extracellular matrix remodeling, a process closely related to tumor invasion and metastasis[28,29].

Our study particularly focused on the heterogeneity of PTC risk factors across different regions. Stratification analysis showed that although SYT1 was the most important feature for PTC diagnosis across all three continents, other key genes differed among regions. This regional heterogeneity may reflect differences in genetic background, environmental exposure, and lifestyle factors, which is consistent with previously reported geographical variations in PTC incidence and clinical characteristics[30–33]. In contrast, factors promoting PTC metastasis exhibited greater consistency across regions, with COL9A1, CYP4F3, and GAD1 identified as important predictors across all three continents, suggesting that PTC metastasis may follow more conserved molecular mechanisms.

Pathway enrichment analysis further revealed potential mechanisms underlying PTC occurrence and metastasis. We found that regulation of hormone levels and cell population proliferation were common pathways involved in both PTC occurrence and metastasis, consistent with the endocrine characteristics and tumor biology of thyroid cancer[34]. Additionally, the

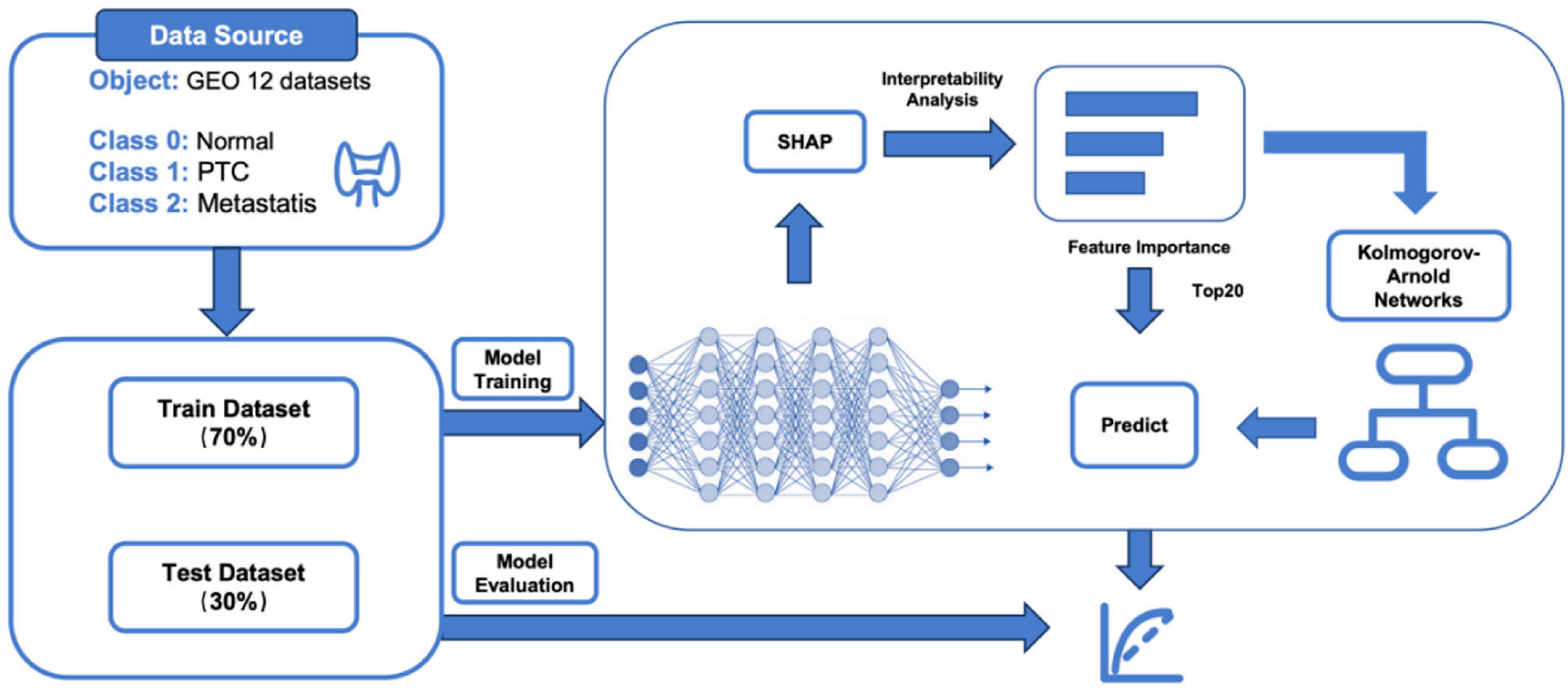

Fig. 7 | Workflow of the study.

enrichment of neural system-related pathways in PTC occurrence and metastasis suggests that neuroendocrine regulation may play an important role in PTC progression, providing new directions for future research.

Another significant contribution of this study is the development of online predictive platforms based on the Streamlit framework, making research findings conveniently applicable to clinical practice. These platforms provide an accessible interface for model inference and individualized feature attribution. At present, they should be regarded as publicly accessible research-use prototypes rather than standalone clinical decision systems. Because the calculators require preprocessed standardized gene-expression inputs and have not yet undergone prospective clinical validation, their value in real-world diagnosis or metastasis risk stratification remains to be established.

The diagnostic models developed in this study were trained using transcriptomic profiles derived from postoperative tumor tissue specimens. In clinical practice, such models may serve as a complementary tool to assist pathologists in confirming the diagnosis of papillary thyroid carcinoma and supporting refined risk stratification. Although the present work did not directly assess the performance of the models on fine-needle aspiration (FNA) samples, the underlying analytical framework is fully compatible with RNA-sequencing data generated from FNA cytology. With the rapid development of low-input RNA-sequencing technologies, it is increasingly feasible to obtain high-quality transcriptomic profiles from routinely collected FNA samples. Our analytical framework may be adaptable to future preoperative applications, including FNA-derived transcriptomic profiling, particularly for indeterminate cytology. However, this possibility remains speculative at present and will require dedicated prospective validation, technical standardization, and workflow assessment before clinical translation. Nevertheless, prospective studies specifically designed to validate performance in FNA-derived transcriptomes will be essential before clinical deployment.

Despite the meaningful findings of this study, several limitations should be acknowledged. First, because the models were developed using retrospectively collected public datasets, the sample size remained limited, particularly for metastatic cases, which may affect model stability and generalizability. Second, although we included geographically diverse cohorts, heterogeneity in platform type, sample processing, and cohort composition may still have influenced the observed performance. Third, this study did not incorporate clinicopathological variables for joint modeling, and therefore potentially informative predictors were not assessed in combination with transcriptomic features. Fourth, the external validation of the metastasis model should be interpreted cautiously because the TCGA-THCA cohort contained only eight annotated metastatic cases, necessitating repeated random undersampling of non-metastatic samples. Finally, the study lacked prospective multicenter validation, and the publicly accessible web calculators are research-use prototypes that require standardized gene-expression inputs rather than direct raw clinical measurements. Accordingly, they should not be used as standalone clinical decision-making tools. Future studies should expand sample size, integrate molecular and clinicopathological variables, and perform prospective multicenter validation to further establish the robustness and clinical utility of these models.

In conclusion, this study successfully developed interpretable artificial intelligence models based on transcriptomics for PTC diagnosis and lymph node metastasis prediction and revealed the heterogeneity of risk factors across different regions. These findings deepen our understanding of transcriptomic features associated with PTC occurrence and lymph node metastasis and provide a basis for the future development of supplementary risk-assessment tools. Their clinical impact should be established through prospective and multicenter validation.

Methods

Study design

This study aims to develop an interpretable artificial intelligence (AI) model based on transcriptomics to predict PTC and cervical lymph node metastasis. The workflow of the study is depicted in Fig. 7.

Data sources

The gene expression data used in this study were sourced from the Gene Expression Omnibus (GEO) database, a repository for high-throughput gene expression data, including microarray and next-generation sequencing data. The detailed information was shown in Table 1. These datasets included a total of 419 samples, with 158 normal samples (37.71%), 203 PTC samples (48.45%), and 58 lymph node metastatic samples (13.84%). The samples were geographically diverse, originating from Asia (24.58%), Europe (47.26%), and America (28.16%). TCGA-THCA was used as the independent external validation set, including 505 primary tumors, 59 normal samples and 8 lymph node metastatic samples.

Data preprocessing

Prior to model development, several preprocessing procedures were applied to ensure the quality and consistency of the transcriptomic data. First, samples that did not correspond to papillary thyroid carcinoma were removed to maintain a biologically homogeneous dataset. Batch effects arising from different sequencing runs or processing batches were corrected using the ComBat algorithm. Outliers in the gene expression matrix were identified

Table 1 | Baseline information of participants in this study

Variables	Total (*n* = 419)	Participants			*P*
		Normal (*n* = 158)	Tumor (*n* = 203)	Metastasis (*n* = 58)	
Continents, *n* (%)					<0.001
America	118 (28.162)	40 (25.316)	78 (38.424)	0 (0.000)	
Europe	198 (47.255)	78 (49.367)	97 (47.783)	23 (39.655)	
Asia	103 (24.582)	40 (25.316)	28 (13.793)	35 (60.345)	
Gse, *n* (%)					<0.001
GSE6004	18 (4.296)	4 (2.532)	14 (6.897)	0 (0.000)	
GSE60542	86 (20.525)	30 (18.987)	33 (16.256)	23 (39.655)	
GSE53157	18 (4.296)	3 (1.899)	15 (7.389)	0 (0.000)	
GSE205733	8 (1.909)	4 (2.532)	4 (1.970)	0 (0.000)	
GSE9115	14 (3.341)	4 (2.532)	10 (4.926)	0 (0.000)	
GSE5364	51 (12.172)	16 (10.127)	0 (0.000)	35 (60.345)	
GSE275666	22 (5.251)	9 (5.696)	13 (6.404)	0 (0.000)	
GSE3678	14 (3.341)	7 (4.430)	7 (3.448)	0 (0.000)	
GSE33630	94 (22.434)	45 (28.481)	49 (24.138)	0 (0.000)	
GSE3467	18 (4.296)	9 (5.696)	9 (4.433)	0 (0.000)	
GSE83520	24 (5.728)	12 (7.595)	12 (5.911)	0 (0.000)	
GSE201365	12 (2.864)	6 (3.797)	6 (2.956)	0 (0.000)	
GSE66783	10 (2.387)	5 (3.165)	5 (2.463)	0 (0.000)	
GSE27155	30 (7.160)	4 (2.532)	26 (12.808)	0 (0.000)	

using a Z-score–based detection method ($|Z| >$ threshold) and removed to prevent extreme values from disproportionately influencing model training. After outlier removal, all gene expression values were standardized using Z-score normalization within each training fold to ensure comparability across samples and to prevent data leakage. These processed and standardized data served as the foundation for all downstream modeling analyses.

Model construction and evaluation

The data were divided into a training set (70%) and a test set (30%). The training set was used to construct the predictive model. The test set was used as an internal validation. Deep neural network (DNN), Gradient Boosting (GB), bootstrap aggregation (Bagging), Adaptive Boosting (AdaBoost), and Logistic Regression (LR) were used to construct the PTC diagnosis model. DNN, Bagging, AdaBoost, K Nearest Neighbors (KNN), Decision Tree (DT) were employed to develop the PTC metastasis predictive model. Model performance was evaluated by area under receiver operating characteristic curve (AUC), accuracy, precision, recall and F1 score.

Undersampling strategy

The dataset exhibited severe class imbalance, for that only eight PTC samples in TCGA-THCA were annotated with lymph-node metastasis. To mitigate this issue for metastasis prediction, we applied a random undersampling strategy. Specifically, from the 505 tumor samples, a subset of non-metastatic samples was randomly selected to match the number of metastatic samples. Three independent balanced datasets were generated through repeated random undersampling, each consisting of eight metastatic and eight non-metastatic samples as shown in Supplementary Table 3. These balanced datasets were then used to assess the generalizability of the metastasis prediction model.

Feature selection

To reduce dimensionality and identify the most informative transcriptomic features, we adopted a two-stage model-based feature selection strategy. In the first stage, deep neural network (DNN) models were trained using the full set of 3481 genes. The model trained on the tumor-versus-normal dataset achieved an AUC of 0.921, while the model trained on the tumor-versus-metastasis dataset achieved an AUC of 0.973, confirming that the global gene-expression profiles contained sufficient discriminative signal. In the second stage, SHapley Additive exPlanations (SHAP) values were computed for each gene to quantify its contribution to the DNN predictions. For each classification task, all genes were ranked according to their mean absolute SHAP values, and the top 20 genes were selected as the final feature set for subsequent modeling. This approach ensured that the selected features were both data-driven and directly linked to model interpretability.

Model interpretation

The optimal deep learning model was interpreted by SHapley Additive exPlanations (SHAP). SHAP global interpretation was illustrated by bar plot and beeswarm plot. SHAP local interpretation was visualized by force plot and waterfall diagrams. Furthermore, Kolmogorov-Arnold Networks (KAN) was investigated to effectively captures complex nonlinear relationships between multi-gene features and categorical outcomes. The optimal PTC diagnostic model and PTC metastasis predictive model were further explained by the ROC curve of KAN, network analysis, expression formulas, linear fitting equation and feature importance rank.

Online platform

The network calculators of the final PTC diagnostic model and PTC metastasis predictive model were developed based on the Streamlit framework, respectively. For both calculators, each gene-expression input is entered as a standardized expression value (unitless z-score) generated using the same preprocessing pipeline as the model-training data, rather than as a raw laboratory measurement. These web calculators were made publicly accessible as research-use prototypes for model demonstration and interpretability visualization. They have not been prospectively validated in clinical workflows and should not be used as standalone tools for clinical diagnosis, risk stratification, or treatment decision-making.

Statistical analysis

All analysis was performed using R V4.4.1 and Python V3.9.12. The DNN model was constructed using "tensorflow" (V1.13.1), and the KAN model was constructed using "pykan" (V0.2.8). Gene Ontology (GO) and the Kyoto Encyclopedia of Genes and Genomes (KEGG) was performed using R ClusterProfile (V3.14.3) and ggplot2 (V3.3.3).

Data availability

The gene expression datasets analyzed in this study are available in the Gene Expression Omnibus (GEO) repository at https://www.ncbi.nlm.nih.gov/geo/ under accession numbers GSE6004, GSE60542, GSE53157, GSE205733, GSE9115, GSE5364, GSE275666, GSE3678, GSE33630, GSE3467, GSE83520, GSE201365, GSE66783, GSE27155. The data used for the external validation of the model generalizability are available in the TCGA-THCA repository at https://portal.gdc.cancer.gov/. All other data supporting the findings are included in the manuscript or supplementary materials. For the online calculators, users should input standardized gene-expression values (unitless z-scores) generated using the same preprocessing pipeline as the model-training data, rather than raw counts, Ct values, TPM/FPKM values, or raw microarray intensities. The publicly accessible web calculators are provided for research demonstration only.

Code availability

Code available at https://github.com/AFS-lunlun/PTC20418 and https://github.com/AFS-lunlun/Streamlit_PTC0418.

Received: 5 July 2025; Accepted: 8 April 2026;

References

1. Sung, H. et al. Global Cancer Statistics 2020: GLOBOCAN estimates of incidence and mortality Worldwide for 36 cancers in 185 countries. *CA Cancer J. Clin.* **71**, 209–249 (2021).
2. La Vecchia, C. et al. Thyroid cancer mortality and incidence: a global overview. *Int. J. Cancer* **136**, 2187–2195 (2015).
3. Haugen, B. R. et al. 2015 American Thyroid Association Management Guidelines for Adult Patients with Thyroid Nodules and Differentiated Thyroid Cancer: The American Thyroid Association Guidelines Task Force on Thyroid Nodules and Differentiated Thyroid Cancer. *Thyroid* **26**, 1–133 (2016).
4. Nikiforov, Y. E. & Nikiforova, M. N. Molecular genetics and diagnosis of thyroid cancer. *Nat. Rev. Endocrinol.* **7**, 569–580 (2011).
5. Scheffel, R. S., Dora, J. M. & Maia, A. L. BRAF mutations in thyroid cancer. *Curr. Opin. Oncol.* **34**, 9–18 (2022).
6. Tuttle, R. M. et al. Estimating risk of recurrence in differentiated thyroid cancer after total thyroidectomy and radioactive iodine remnant ablation: using response to therapy variables to modify the initial risk estimates predicted by the new American thyroid association staging system. *Thyroid* **20**, 1341–9 (2010).
7. Kim, T. H. et al. Refining dynamic risk stratification and prognostic groups for differentiated thyroid cancer with TERT promoter mutations. *J. Clin. Endocrinol. Metab.* **102**, 1757–1764 (2017).
8. Filetti, S. et al. ESMO Clinical Practice Guideline update on the use of systemic therapy in advanced thyroid cancer. *Ann. Oncol.* **33**, 674–684 (2022).
9. Liu, Z. et al. Machine learning-based integration develops an immune-derived lncRNA signature for improving outcomes in colorectal cancer. *Nat. Commun.* **13**, 816 (2022).
10. Liang, Y., Zhang, H., Song, X. & Yang, Q. Metastatic heterogeneity of breast cancer: Molecular mechanism and potential therapeutic targets. *Semin. Cancer Biol.* **60**, 14–27 (2020).
11. Ennab, M. & Mcheick, H. Enhancing interpretability and accuracy of AI models in healthcare: a comprehensive review on challenges and future directions. *Front Robot AI* **11**, (2024).
12. Hassija, V. et al. Interpreting black-box models: a review on explainable artificial intelligence. *Cogn. Comput* **16**, 45–74 (2024).
13. Antwarg, L., Miller, R. M., Shapira, B. & Rokach, L. Explaining anomalies detected by autoencoders using Shapley Additive Explanations. *Expert Syst. Appl.* **186**, 115736 (2021).
14. Ekanayake, I. U., Meddage, D. P. P. & Rathnayake, U. A novel approach to explain the black-box nature of machine learning in compressive strength predictions of concrete using Shapley additive explanations (SHAP). *Case Stud. Constr. Mater.* **16**, e01059 (2022).
15. Liu, Z. et al. KAN: Kolmogorov-Arnold Networks. (2024).
16. Hou, F. et al. Development and validation of an interpretable machine learning model for predicting the risk of distant metastasis in papillary thyroid cancer: a multicenter study. *EClinicalMedicine* **77**, 102913 (2024).
17. Schneider, D. F. & Chen, H. New developments in the diagnosis and treatment of thyroid cancer. *CA Cancer J. Clin.* **63**, 373–394 (2013).
18. Chen, D. W., Lang, B. H. H., McLeod, D. S. A., Newbold, K. & Haymart, M. R. Thyroid cancer. *Lancet* **401**, 1531–1544 (2023).
19. Courtney, N. A., Bao, H., Briguglio, J. S. & Chapman, E. R. Synaptotagmin 1 clamps synaptic vesicle fusion in mammalian neurons independent of complexin. *Nat. Commun.* **10**, 4076 (2019).
20. Suo, H., Xiao, N. & Wang, K. Potential roles of synaptotagmin family members in cancers: recent advances and prospects. *Front Med. (Lausanne)* **9**, (2022).
21. Shi, J. et al. Synaptotagmin 1 suppresses colorectal cancer metastasis by inhibiting ERK/MAPK signaling-mediated tumor cell pseudopodial formation and migration. *Cancers (Basel)* **15**, 5282 (2023).
22. Redondo, P. C. et al. Extended synaptotagmins 1 and 2 are required for store-operated calcium entry, cell migration and viability in breast cancer cells. *Cancers (Basel)* **16**, 2518 (2024).
23. Luo, M.-L. et al. An ADAM12 and FAK positive feedback loop amplifies the interaction signal of tumor cells with extracellular matrix to promote esophageal cancer metastasis. *Cancer Lett.* **422**, 118–128 (2018).
24. Wang, R. et al. Hypoxia-inducible factor-dependent ADAM12 expression mediates breast cancer invasion and metastasis. *Proc. Natl. Acad. Sci. USA* **118**, (2021).
25. Duhachek-Muggy, S. et al. Metalloprotease-disintegrin ADAM12 actively promotes the stem cell-like phenotype in claudin-low breast cancer. *Mol. Cancer* **16**, 32 (2017).
26. Zhu, H. et al. Elevation of ADAM12 facilitates tumor progression by enhancing metastasis and immune infiltration in gastric cancer. *Int J. Oncol.* **60**, 51 (2022).
27. Frohlich, C. et al. Molecular profiling of ADAM12 in human bladder cancer. *Clin. Cancer Res.* **12**, 7359–7368 (2006).
28. Maftouh, M. & Avan, A. 102P The predictive potential value of collagen type IX alpha 1 chain in colorectal cancer: integrative analysis using RNA/DNA sequencing and pan-cancer studies. *ESMO Open* **10**, 104263 (2025).
29. Singh, M. K., Singh, V. & Kumar, A. 101P Survival analysis and clinicopathological correlation with molecular classification in BCG-nonresponsive Ta-T1 bladder cancer. *ESMO Open* **10**, 104262 (2025).
30. Lim, H., Devesa, S. S., Sosa, J. A., Check, D. & Kitahara, C. M. Trends in thyroid cancer incidence and mortality in the United States, 1974-2013. *JAMA* **317**, 1338 (2017).
31. Davies, L. & Welch, H. G. Increasing incidence of thyroid cancer in the United States, 1973-2002. *JAMA* **295**, 2164 (2006).
32. Kitahara, C. M., Pfeiffer, R. M., Sosa, J. A. & Shiels, M. S. Impact of overweight and obesity on US papillary thyroid cancer incidence trends (1995–2015). *JNCI: J. Natl. Cancer Inst.* **112**, 810–817 (2020).
33. Sweis, N. W. G. et al. Geographic variation in the association between Hashimoto's thyroiditis and Papillary thyroid carcinoma, a meta-analysis. *Endocrine* **81**, 432–449 (2023).
34. Xing, M. Molecular pathogenesis and mechanisms of thyroid cancer. *Nat. Rev. Cancer* **13**, 184–199 (2013).

Acknowledgements

We thank Guoyu Tan and members of the Lun Lun Research Institute of AI FOR SCIENCE for technical support and suggestions that improved the manuscript and experiments. This study was supported by Liaoning Provincial Science and Technology Plan Joint Program, 2024-MSLH-583.

Author contributions

Z.Z. and H.L. helped in writing of the original draft. Z.z. and G.T. helped in formal analysis. X.L. and Y.Z. helped in and data curation and conceptualization.

Competing interests

The authors declare no competing interests.

Additional information

Supplementary information The online version contains supplementary material available at https://doi.org/10.1038/s41746-026-02649-8.

Correspondence and requests for materials should be addressed to Yang Zhao or Xun Liu.

Reprints and permissions information is available at http://www.nature.com/reprints

Publisher's note Springer Nature remains neutral with regard to jurisdictional claims in published maps and institutional affiliations.

Open Access This article is licensed under a Creative Commons Attribution 4.0 International License, which permits use, sharing, adaptation, distribution and reproduction in any medium or format, as long as you give appropriate credit to the original author(s) and the source, provide a link to the Creative Commons licence, and indicate if changes were made. The images or other third party material in this article are included in the article's Creative Commons licence, unless indicated otherwise in a credit line to the material. If material is not included in the article's Creative Commons licence and your intended use is not permitted by statutory regulation or exceeds the permitted use, you will need to obtain permission directly from the copyright holder. To view a copy of this licence, visit http://creativecommons.org/licenses/by/4.0/.

© The Author(s) 2026
